# Diversity of immune checkpoints in cancer immunotherapy

**DOI:** 10.3389/fimmu.2023.1121285

**Published:** 2023-03-07

**Authors:** Zhangyan Guo, Rui Zhang, An-Gang Yang, Guoxu Zheng

**Affiliations:** ^1^ State Key Laboratory of Cancer Biology, Department of Immunology, Fourth Military Medical University, Xi’an, China; ^2^ State Key Laboratory of Cancer Biology, Department of Biochemistry and Molecular Biology, Fourth Military Medical University, Xi’an, China

**Keywords:** immune checkpoint, immunotherapy, T cell, NK cell, macrophage

## Abstract

Finding effective treatments for cancer remains a challenge. Recent studies have found that the mechanisms of tumor evasion are becoming increasingly diverse, including abnormal expression of immune checkpoint molecules on different immune cells, in particular T cells, natural killer cells, macrophages and others. In this review, we discuss the checkpoint molecules with enhanced expression on these lymphocytes and their consequences on immune effector functions. Dissecting the diverse roles of immune checkpoints in different immune cells is crucial for a full understanding of immunotherapy using checkpoint inhibitors.

## 1 Introduction

**Figure 1:**
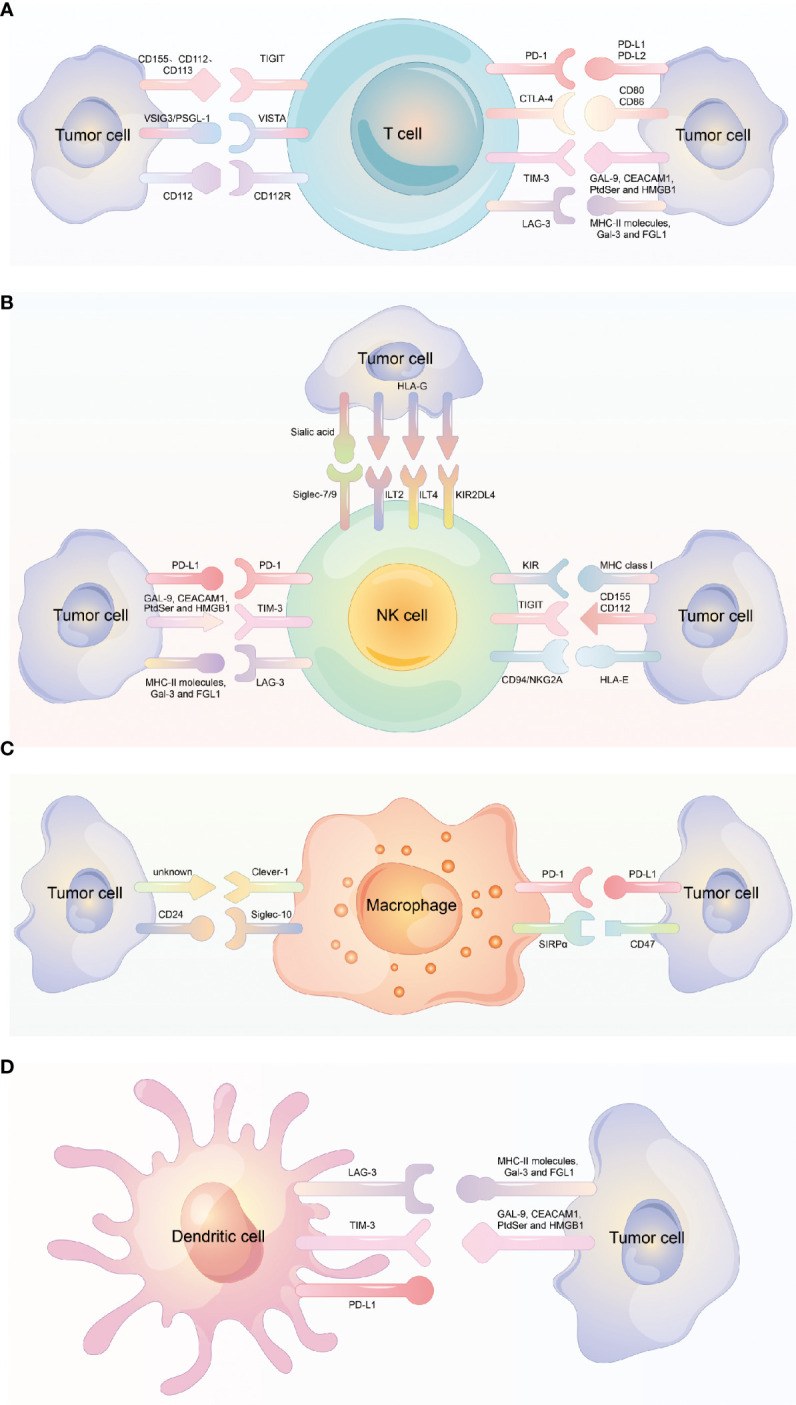
Different immune checkpoint molecules expressed on different immune cells. **(A)** Different immune checkpoint molecules expressed on T cell and the corresponding ligand molecules expressed on tumor cells. **(B)** Different immune checkpoint molecules expressed on NK cell and the corresponding ligand molecules expressed on tumor cells. **(C)** Different immune checkpoint molecules expressed on Macrophage and the corresponding ligand molecules expressed on tumor cells. **(D)** Different immune checkpoint molecules expressed on dendritic cell and the corresponding ligand molecules expressed on tumor cells.

It now appears that immunotherapies can elicit durable antitumor responses in metastatic cancer. These immunotherapies include adoptive cell therapy (ACT) and checkpoint inhibitor therapies (1). In particular, recent studies have confirmed that targeting immune checkpoint pathways has remarkable clinical efficiency across several tumor types (2). Immune checkpoint molecules are mainly expressed on immune cells and can maintain immunological homeostasis. Under normal physiological conditions, they can inhibit and prevent immune cells from killing tumor cells (3). In the past few years, studies have mainly focused on finding new immune checkpoint molecules expressed on T cells, which can effectively restore the exhaustion of T cells when blocked. The immune checkpoint targets that have been validated clinically include CTLA4 and PD-1, and many new candidates are being discovered and will undergo clinical evaluation (4). In addition to T cells, Nature Killer cells also express immune checkpoints, but the consequences of these checkpoints on NK cells’ functions are much less explored (5). Recently, literature has shown that macrophage-centered blockade of immune checkpoints represents promising therapeutic avenues (6). In this review, we will discuss recent advances in knowledge regarding the diversity of immune checkpoints expressed on different immune cells and their relationships with cancer immunotherapy (**Figure 1**).

## 2 Checkpoint immunotherapy based on T cells

In the last few decades, the function of tumor-infiltrating lymphocytes (TILs), especially the cytotoxic CD8^+^ T cells and other subgroups of T cells, such as CD4^+^ T cells and Tregs on tumor progression and patient prognosis have been deeply explored (7–9).

In immunological homeostasis, the engagement of T-cell antigen receptors (TCRs) with antigenic peptides can result in the activation and proliferation of T cells (10). To prevent overreaction and autoimmunity, inhibitory receptors are upregulated on T cells and other immune cells. These inhibitory receptors are also called immune checkpoints. Because of the presence of the immunoreceptor tyrosine-based inhibitory motif (ITIM), immune checkpoints can induce inhibitory signals in inhibitory receptor-expressing immune cells (11).

In the immunosuppressive tumor microenvironment, tumor cells make use of the overexpression of inhibitory receptors on immune cells to avoid immune clearance (12). The expression of immune checkpoints can lead to T-cell exhaustion, which is defined by a decline in T-cell proliferation and reduced T-cell function. To date, immune checkpoints that have been explored for their expression by T cells include PD-1 (programmed cell death protein-1), CTLA-4 (cytotoxic T-lymphocyte-associated protein-4), TIM-3 (mucin-domain containing-3), LAG-3 (lymphocyte-activation gene-3), and T cell immunoglobulin and ITIM domain (TIGIT), among others (13).

### 2.1 PD-1

PD-1 (CD279) is a coinhibitory receptor that is extensively expressed on T cells, NK cells (natural killer cells), and B cells. In particular, PD-1 is expressed on activated T cells at high levels and is considered to be involved in immune tolerance (14). There are two ligands for PD-1, known as PD-L1 and PD-L2, which have low expression in normal tissue but abnormal expression in some tumor types. For example, it has been reported that the expression of PD-L1 is upregulated in melanoma, non-small-cell lung cancer, breast cancer, and squamous cell head and neck cancer (15).

PD-1^+^ T-cell exhaustion was originally studied in murine models and then extended to human infection and cancer (16). In chronic viral infections, CD8^+^ T cells are in a state of dysfunction and have abnormal expression of PD-1. Se Jin Im et al. found that in a mouse model chronically infected with lymphocytic choriomeningitis virus, a population of virus-specific CD8^+^ T cells proliferated after PD-1 blockade, and this proliferative burst occurred only in this type of CD8^+^ T cell (17). Tim Wartewig et al. found that mono- and biallelic deletions of PDCD1, which encodes PD-1, are recurrently observed in human T-cell lymphomas with frequencies of up to 30%, indicating high clinical relevance; these findings imply that PD-1 is a potent haploinsufficient tumor suppressor in T-cell lymphomas (18). In a study of colorectal cancer, Xiao Albert Zhou et al. identified a major PD-1-associated protein, KLHL22, that can mediate the degradation of PD-1 before its transport to the cell surface. They found that the expression of KLHL22 was markedly decreased in tumor-infiltrating T cells from colorectal cancer patients and suggested the therapeutic potential of 5-FU (which could increase PD-1 expression by inhibiting the transcription of KLHL22) in combination with anti-PD-1 in colorectal cancer patients (19).

Based on previous research, new strategies have emerged that target PD-1 or PD-L1 and block them; as a result, T-cell function is successfully reinvigorated (20). Along these lines, antibodies targeting the PD-1/PD-L1 axis have been used for various tumors. For example, Alexander C Huang et al. found that neoadjuvant anti-PD-1 treatment is effective against high-risk resectable stage III/IV melanoma (21). Edward B Garon et al. assessed the efficacy and safety of PD-1 inhibition with pembrolizumab in patients with advanced non-small-cell lung cancer enrolled in a phase 1 study and found that a blocking antibody targeting PD-1 had an obvious antitumor effect in NSCLC patients and an acceptable side-effect profile (22). Fan Zhang et al. performed scRNA-seq analysis on 3110 peripheral T cells of NSCLC patients before and after the initiation of PD-1 blockade and found a higher cytotoxic activity in tumor-related CD4^+^ T-cell clones than in CD8^+^ T-cell clones (23). In a prognostic analysis of advanced renal cell carcinoma, the investigator assessed the efficacy and safety of nivolumab treatment *versus* everolimus treatment over a 3-year follow-up and found that nivolumab treatment was more effective and safer than everolimus (24). Two phase III clinical trials (CheckMate 141 and KEYNOTE 040) analyzed the overall survival (OS) of patients with recurrent or metastatic head-and-neck squamous cell carcinoma (HNSCC) and found that anti-PD-1 monotherapy improved the therapeutic effects of platinum chemotherapy (25, 26).

Although a promising therapeutic effect using a PD-1 blocking antibody was observed in those tumor patients, some patients did not respond to this blocking antibody blocking, or it had limited effects. This implies that there are other inhibitory pathways involved in T-cell dysfunction.

### 2.2 CTLA-4

CTLA-4 (cytotoxic T-lymphocyte-associated protein 4), also known as CD152, is a protein receptor mainly expressed on T cells that was first identified as a second receptor for the T-cell costimulatory legend B7 and later discovered to be a negative regulator of T-cell activation (27–29). In naïve T cells, the expression of CTLA4 is low, but in phases of TCR engagement and activation, CTLA4 can be rapidly upregulated in both CD4 helper T cells and CD8 effector T cells, while its upregulation is obvious in helper T cells (30). CTLA4 has two ligands, CD80 and CD86, also called B7-1 and B7-2, which can also be recognized by CD28, a T-cell costimulatory protein that is homologous to CTLA4. However, for both ligands, CTLA4 has higher affinity and avidity than CD28, implying that it is an antagonist of CD28-mediated costimulation (31, 32). This mechanism suggests that the CD28/CTLA4 regulatory form can act as a rheostat in T-cell activation.

In mouse models, anti-CTLA4 antibody treatment initially resulted in the rejection of tumors, including preestablished tumors; furthermore, the rejection resulted in immunity to a secondary exposure to tumor cells (33). During the subsequent development of clinical immunotherapy, two CTLA-4 blockade antibodies, ipilimumab and tremelimumab, have been tested in many types of human tumors, and their treatment efficacy has been reported in melanoma (34, 35), non-small-cell lung cancer (36), mesothelioma (37), prostate cancer (38), breast cancer (39) and urothelial cancer (40). Despite the promising therapeutic effects, a broad range of immune-related adverse events (irAEs) occurring in the skin, gastrointestinal tract, liver and endocrine organs have been reported in some trials, with an incidence of 60-65% (41). A landmark clinical trial called the CheckMate 067 clinical trial (ClinicalTrials.gov NCT01844505) used a combination CPI therapy with an anti-CTLA-4 antibody and an anti-PD-1 antibody This study was carried out on 945 patients with stage III or IV melanoma and evaluated the median overall survival under treatment with nivolumab plus ipilimumab or with nivolumab or ipilimumab monotherapy. Although the results showed that the OS appeared to be improved in the combination treatment cohort compared with the single-treatment cohorts, the trial did not have sufficient power to show a significant difference between the two nivolumab-containing groups, and the incidence of adverse events was increased in the combination therapy cohort in this trial (42, 43).

Additionally, while CTLA4 is expressed at high levels on Tregs and although an important role of conventional T-cell CTLA4 in self-tolerance has been reported, CTLA4 blockade therapy combined with Treg depletion has led to considerable success in tumor treatment as well as autoimmune disease treatment (41). Therefore, more research should be conducted to reveal the pros and cons of CTLA4 blockade immunotherapy.

### 2.3 TIM-3

TIM-3 (T-cell immunoglobulin and mucin domain containing-3), a member of the TIM family is a coinhibitory receptors. It is expressed on IFN-γ-producing T helper 1 CD4^+^ and CD8^+^ T cells and Th17 cells (44). The expression of TIM-3 is regulated by antigenic stimulation and proinflammatory cytokines (45). In early studies, TIM-3 was reported to have an inhibitory function, suppressing effector Th1 responses in EAE and type I diabetes in a mouse model, and the use of an anti-TIM-3 antibody was reported to lead to disease exacerbation in EAE (46). In subsequent studies, the overexpression of TIM-3 has been found to be correlated with T-cell dysfunction and T-cell exhaustion (47). The role of TIM-3 as a suppressive receptor that regulates T-cell activity in some chronic viral infections, such as HIV-1, HBV and HCV infections, has been reported (48–50). In the tumor microenvironment, TIM-3 has also been found to be expressed on CD8^+^ TILs (tumor-infiltrating leukocytes), which is closely associated with PD-1 expression. Specifically, the expression patterns of TIM-3 and PD-1 indicate the degree of T-cell exhaustion; for example, in mice bearing solid tumors, TIM-3^+^PD-1^+^ TILs exhibit the most severely exhausted phenotype, as defined by failure to proliferate and produce cytokines. Additionally, high expression of TIM-3 on CD8^+^ T cells has been found to be correlated with poor prognosis in certain types of cancers, and blockade of TIM-3 combined with anti-PD-1 antibody treatment has been confirmed to be more effective than blockade of either molecule alone in antitumor immunotherapy (51–53). In a study on medullary thyroid carcinoma (MTC) in 200 MTC patients, TIM-3 positivity was 48%, and TIM-3 expression was positively correlated with PD-1 and CTLA-4 expression. Log-rank tests and multivariate Cox analyses both indicated that TIM-3, CTLA-4 and PD-1/PD-L1 coexpression were associated with poor structural recurrence-free survival (54).

### 2.4 LAG-3

LAG-3, lymphocyte activation gene 3, is a cell surface protein belonging to the immunoglobulin superfamily that is expressed on CD4+ and CD8+ T cells (55), NK cells (56), B cells and plasmacytoid dendritic cells (57). It is a coinhibitory transmembrane receptor whose ligands are MHC class II and FGL1, and interaction with the ligands can negatively regulate the activation of T cells (58, 59), similar to the case for CTLA4 and PD-1 (60, 61). In particular, LAG-3 has a synergistic effect with PD-1 to regulate immune responses (62). In clinical immunotherapy, a LAG-3 Ig fusion protein named IMP321 was first used in advanced renal cell carcinoma patients and resulted in reduced tumor growth and improved progression-free survival (63). When LAG-3 blockade antibody (BMS-986016) and nivolumab (a PD-1 antibody) were used in combination in melanoma patients, the initial resistance when only blocking of the PD-1/PD-L1 axis was converted (64). In addition, many types of human tumors present aberrant expression of LAG-3, which correlates with poor outcomes (65–69). Kosaku Mimura et al. evaluated the distribution of different inhibitory ligands in 365 GC patients and found coexpression of inhibitory ligands for PD-1, Tim-3 and Lag-3 in the largest proportion (34.7%). Their findings suggest that the expression of inhibitory ligands for Tim-3 and Lag-3 on GC cells serve as potential predictive biomarkers of the response to anti-PD-1 therapy (70).

### 2.5 TIGIT

TIGIT, T-cell immunoglobulin and ITIM domain, belongs to the immunoglobulin superfamily and is also a T-cell coinhibitory receptor. It is expressed on CD4^+^ memory and regulatory T cells, CD8^+^ T cells and NK cells. To date, the ligands that have been discovered to be recognized by TIGIT are CD155 (PVR or poliovirus receptor), CD112 (PVRL2) and CD113 (PVRL3, NECTIN-3), of which CD155 has the highest affinity for TIGIT (71). TIGIT has been implicated in tumor immunosurveillance, and its role is analogous to that of PD-1 in tumor immunosuppression because it is overexpressed in tumor antigen-specific CD8^+^ T cells and CD8^+^ TILs and is often coexpressed with PD-1. Therefore, co-blockade of the two checkpoint molecules can enhance the antitumor efficacy of single blockade (72).

### 2.6 VISTA

VISTA, V-domain Ig-containing suppressor of T-cell activation, also belongs to the transmembrane Ig superfamily (73). It is part of the B7 family and is mainly expressed on T cells and CD11b^+^ antigen-presenting cells (APCs)/myeloid cells (74). It has been reported that VISTA can act as both a receptor and a ligand on T cells and that it functions as an inhibitor to maintain immune tolerance (75). In tumor-infiltrating lymphocytes, VISTA is overexpressed, especially in myeloid-derived suppressor cells and regulatory T cells. Recently, it has been reported to be highly expressed in human ovarian and endometrial cancers. The abnormal expression of VISTA in tumor cells suppresses T-cell proliferation and cytokine production *in vitro* and decreases the tumor infiltration of CD8^+^ T cells *in vivo*. VISTA blockade prolongs the survival of tumor-bearing mice (76). In a study on oropharyngeal squamous cell carcinoma (OPSCC) including 241 tumor tissues aiming to describe the expression of LAG-3, Tim-3, and VISTA in the TME of OPSCC, immunohistochemistry showed that 168 OPSCC samples stained positive for VISTA. The results also revealed that CD8^+^ T cells were significantly associated with LAG-3, Tim-3 and VISTA expression (*p* < 0.001, *p* < 0.001, *p* = 0.007), so immune checkpoint therapy targeting LAG-3, Tim-3, and/or VISTA could be a promising treatment strategy, especially for HPV-related OPSCC (77).

### 2.7 Siglec-15

Siglec-15, short for sialic acid-binding immunoglobulin-like lectin 15, belongs to the Siglec gene family because of its sialic acid-binding immunoglobulin-type lectin structure (78). Originally, Siglec-15 was mainly reported to play roles in osteoclast differentiation and bone remodeling (79, 80). Recently, Wang et al. identified Siglec-15 as a potent immunosuppressive molecule. In their study, using a newly developed genome-scale T-cell Activity Array, they identified that the expression of Siglec-15 was upregulated in many human cancer cells and tumor-infiltrating myeloid cells, while under normal physiological conditions, it was limited to cells in the myeloid lineage. In particular, its expression was mutually exclusive with that of B7-H1 in cancer cells and could be regulated by M-CSF and IFN-γ. In thorough *in vitro* and *in vivo* experiments, Siglec-15 was confirmed to suppress antigen-specific T-cell responses and impair antitumor immunity. Conversely, a Siglec-15-blocking mAb reversed T-cell suppression and promoted tumor immunity in multiple tumor models (81). Siglec-15 has unique molecular features compared with those of many other known checkpoint inhibitory ligands; it shows mutually exclusive expression with PD-L1, which suggests that it plays a key role in tumor escape in PD-L1-negative patients. As a new player in cancer immunotherapy, siglec-15 may have potential applications in anti-PD-1/PD-L1-resistant patients (82). Collectively, the evidence suggests that Siglec-15 is an attractive target for cancer immunotherapy.

### 2.8 CD112R

CD112R is a poliovirus receptor-like protein and has been described as a new coinhibitory receptor for human T cells that can interact with CD112 with higher affinity than CD226 and TIGIT. Recently, it has also been reported to be expressed in subpopulations of NK cells (83). Zhu et al. reported that CD112 is expressed on DCs and many tumor cells and mediates the interaction of CD112R with DCs and tumor cells. When the interaction between CD112R and CD112 is disrupted, human T-cell function is enhanced. These results imply that the CD112R/CD112 axis is a new checkpoint in human T cells (84).

## 3 Checkpoint immunotherapy based on NK cells

Natural killer (NK) cells are involved in innate immunity and play a significant role in immunological surveillance against various infections and malignant transformation. Unlike that of T cells, the activation of NK cells does not require prior sensitization, and the NK cell function is determined by the balance of a series of activated and inhibitory receptors expressed on the cell surface (85, 86). In the tumor microenvironment, tumor cells often downregulate the expression of major histocompatibility complex (MHC) class I to escape killing by T cells, nevertheless, these “missing self” tumor cells become more susceptible to the immunosurveillance executed by NK cells. Based on these intrinsic properties and accumulating evidence that defects in NK-cell function and number are often associated with viral infections and tumorigenesis (87), increased attention has been given to NK-cell-based immunotherapy to compensate for the lack of T cell immunotherapy.

### 3.1 KIRs

Killer-cell immunoglobulin-like receptors (KIRs) are a family of type I transmembrane glycoproteins that are expressed on NK cells and a minority of T cells (88). KIRs have dual functions: they can inhibit NK-cell cytotoxicity by interacting with MHC class I molecules but can also activate cytotoxic activity as activating receptors (89). KIR family members have many haplotypes because of their polymorphic genes, such as KIR2DL1 and KIR3DL2, which are named by the number of extracellular immunoglobulin domains and by the length of the cytoplasmic domain they express (90). KIR inhibitory receptors conduct inhibitory signals through the ITIM, which is located in their long cytoplasmic domain. Based on the “missing self” theory, the humanized antagonistic antibody lirilumab (IPH2102), which can target inhibitory KIRs such as KIR2DL1-3 and KIR2DS1-2, has been used in clinical immunotherapy studies (91). Although the use of lirilumab has been shown to promote NK-cell cytotoxicity toward multiple myeloma, lymphoma and leukemia in preclinical studies, its efficacy in some phase I or II trials on multiple myeloma and acute myeloid leukemia was not as good as expected (92–94). Another mAb targeting KIR2DL1/2/3, IPH2102, has failed to exert impressive clinical effects in patients with multiple myeloma (MM) as monotherapy, but when combined with lenalidomide in a dual immunotherapy for MM patients, it has been reported to achieve a median progression-free survival of 24 months, suggesting the promise of combination therapy (95).

### 3.2 NKG2A

NKG2 belongs to the C-type lectin-like receptor superfamily and has seven types, NKG2A, NKG2B, NKG2C, NKG2D, NKG2E, NKG2F and NKG2H. NKG2 is expressed on NK cells and acts as an activating receptor or inhibitory receptor when dimerized with other molecules. CD94/NKG2A forms a heterodimeric receptor and plays an inhibitory role on both T cells and NK cells by interacting with HLA-E, which is upregulated in many tumors (96, 97). Pascale André et al. reported that the use of an NKG2A blocking antibody, monalizumab, can enhance NK-cell effector functions against various tumor cells and can rescue CD8^+^ T-cell function in combination with PD-x axis blockade (98). Takahiro Kamiya et al. constructed NKG2A-null NK cells in which NKG2A expression was abrogated and found that they had increased cytotoxicity against HLA-E-expressing tumor cells. In immunodeficient mice, NKG2A-null NK cells showed an enhanced antitumor effect against HLA-E-expressing tumors (99). In an *in vivo* study on cancer vaccination using mouse tumor models, the impact of therapeutic vaccines was greatly potentiated by disruption of the NKG2A/Qa-1^b^ (conserved ortholog of HLA-E) axis even in a PD-1-refractory mouse model. However, in this research, the blockade therapy affected CD8 T cells, not NK cells. These findings indicate that NKG2A-blocking antibodies might improve clinical responses to therapeutic cancer vaccines (100). Overall, blockade of the NKG2A axis represents a promising therapeutic approach, but monalizumab monotherapy or combination therapy with another blocking antibody (cetuximab or durvalumab) is still under investigation, and more trials are needed.

### 3.3 TIGIT

As mentioned above, TIGIT is expressed on some NK cells and can interact with its ligands CD155 and CD112, which are expressed on many tumor cells (71). The binding of TIGIT with its ligands has been reported to result in an inhibitory signal and downregulate NK-cell functions. Qing Zhang et al. reported that TIGIT was associated with NK-cell exhaustion in mouse models and in patients with colon cancer. In mice bearing tumors, including colon tumors, breast tumors and chemically induced fibrosarcomas, treatment with an mAb to TIGIT induced tumor growth inhibition and tumor volume reduction and prevented NK-cell exhaustion. In addition, blockade of TIGIT resulted in potent tumor-specific T-cell immunity in an NK-cell-dependent manner and exerted a synergistic effect with an mAb blocking PD-1 (101).

### 3.4 PD-1

In addition to being expressed in T cells as mentioned above, PD-1 has also been reported to be expressed in human NK cells from healthy donors and cancer patients and to have an inhibitory effect on NK-cell function (102, 103). Joy Hsu et al. reported that blockade of the PD-1/PD-L1 axis can elicit a strong NK-cell response, which is essential for the therapeutic effect, and implied the importance of PD-1 in inhibiting NK-cell responses *in vivo* and of the coordinating roles of T cells in PD-1/PD-L1 blockade immunotherapy (104). Wenjuan Dong et al. found that some tumors can induce PD-L1 expression on NK cells *via* AKT signaling and that an anti-PD-L1 mAb can directly act on PD-L1^+^ NK cells to combat PD-L1- tumors *via* a p38 pathway. Their findings reveal a PD-1-independent mechanism of antitumor efficacy through PD-L1^+^ NK cells that is activated with an anti-PD-L1 mAb (105).

### 3.5 TIM-3

The expression of TIM-3 is extensive in immune cells, as mentioned above. In addition to T cells, TIM-3 is constitutively expressed on resting human NK cells and is upregulated upon activation (106). The transcriptional levels of TIM-3 are higher in NK cells than in other lymphocytes, and TIM-3 can serve as a maturation marker. Antibodies that crosslink TIM-3 suppress NK-cell-mediated cytotoxicity, indicating that the function of NK cells may be negatively regulated by the interaction of TIM-3 with its cognate ligands, which are expressed on target cells (107). TIM-3 is upregulated in peripheral NK cells of patients with gastric cancer, lung adenocarcinoma and melanoma, while it is upregulated in tumor-infiltrating NK cells of gastrointestinal stromal tumors. This abnormal expression of TIM-3 on NK cells often predicts a poor prognosis, especially in melanoma and lung adenocarcinoma, but blockade of TIM-3 reverses NK-cell exhaustion and improves NK-cell-mediated cytotoxicity (108–111).

### 3.6 LAG-3

LAG-3 is an inhibitory receptor that is upregulated on activated T cells and NK cells, as mentioned above. It is homologous to CD4 but has a greater affinity for MHC class II molecules; additionally, LAG-3 can bind to LSECtin and FGL1, which are expressed by some tumor cells (112). Unlike in T cells, the function of LAG-3 in NK cells is not clear. Although previous studies have not found that blockade of LAG-3 on human NK cells can influence NK-cell cytotoxicity (113), one study reported that patients with HIV have lower expression of LAG-3 along with other inhibitory molecules involved in viral control, such as PD-1 and TIM-3, than individuals in a low-risk population or progressors (114). IMP321, a soluble recombinant LAG-3-Ig fusion protein, has been reported to induce NK cells to produce IFN-γ and/or TNF-α in healthy donors in an ex vivo short-term experiment, but in metastatic cancer patients, the values are reduced (5, 115). In clinical trials, many anti-LAG-3 monoclonal antibodies have been analyzed either as monotherapies or in combination with other checkpoint-blocking antibodies, such as anti-PD-1 mAb, for the immunotherapy of solid tumors and hematologic malignancies. Two examples are relatlimab (BMS-986016) (NCT01968109) and LAG525 (NCT02460224). However, further work on the effect of LAG-3 on NK cells needs to be explored (116).

### 3.7 Siglec-7/9

Siglecs, sialic acid-binding immunoglobulin-type lectins, are a subset of the I-type lectins that bind sialic acid and are mainly expressed on the surfaces of immune cells, including neutrophils, eosinophils, monocytes, macrophages, NK cells, dendritic cells, mast cells, B cells and T cells (117). To date, the siglec receptor family comprises 15 members that vary in their expression patterns and in the specificity of ligand binding. Among the family members, siglec-7 and siglec-9 are reported to be mainly expressed on NK cells and to transport inhibitory signals through the ITIM motifs in their cytoplasmic tails (118). Many studies have reported that changes in sialic acid are correlated with tumorigenesis and cancer progression (119). Therefore, siglec-sialic acid interactions may play an important role in modulating the immune response and can be targeted as useful checkpoints (120). In human cancer, siglec-9 has been found to be upregulated in peripheral NK cells, mainly in CD56^dim^CD16^+^ NK cells. In an *in vitro* study, blockade of siglec-7 and siglec-9 using Fab fragments increased the cytotoxicity of NK cells against tumor cells, and in an *in vivo* mouse model, sialoglycan-dependent NK-cell inhibition led to the killing of tumor cells (118). In a recent study, Itziar Ibarlucea-Benitez et al. investigated the impacts of siglec-7 and siglec-9 on tumor progression using a humanized immunocompetent murine model and found reduced tumor burden when using Fc-engineered anti–Siglec-7 and anti–Siglec-9 blocking antibodies. This effect may have been mediated by prevention of macrophage polarization into tumor-associated macrophages and thus reprogramming of the immune-suppressive tumor microenvironment (121). In addition, Siglec-9 has been found to be upregulated on tumor-infiltrated CD8+ T cells in non-small-cell lung cancer and ovarian and colorectal cancers, and other inhibitory receptors, such as PD-1, are also coexpressed by T cells expressing siglec-9, implying that combination with other immune checkpoint inhibitors could be used for coinhibition in immunotherapy (122).

### 3.8 HLA-G

Human leukocyte antigen (HLA)-G is a nonclassical MHC-I molecule that was initially found to be expressed in pregnancies by cells of the trophoblast at the maternal–fetal interface and acts as a mediator of immune tolerance because it protects the fetus from NK-cell-mediated lysis (123, 124). To date, seven isoforms have been found, including HLA-G1 to HLA-G7, some of which are membrane-bound molecules and some of which are soluble forms. Under normal physiological conditions, the expression of HLA-G is restricted to immune-privileged organs, but it is upregulated in some immune-mediated diseases, such as viral infections and cancer. By interacting with different receptor molecules on different immune cells, HLA-G exerts several immunomodulatory effects. In NK cells, the inhibitory receptors ILT2 and ILT4 are responsible for the HLA-G-mediated inhibitory effect (125). One study has found that these two inhibitory receptors are broadly expressed on T cells, B cells and dendritic cells, implying the immunosuppressive effect of HLA-G on these cells (126). The abnormal expression of HLA-G in different cancers is associated with poor clinical outcomes in patients, so increasing attention has been given to HLA-G as an immune checkpoint in cancer (127). Numerous studies have reported that the expression of HLA-G in ovarian carcinoma, hepatocellular carcinoma, glioma and renal cell carcinoma inhibits NK cell-mediated cytolysis of these cancer cells but that this inhibition can be reversed by the use of specific antibodies targeting HLA-G or its receptors. In addition, the modulation of cytokine secretion by sHLA-G/ILT2 binding and the different immunosuppressive functions of HLA-G on T cells, B cells, macrophages, dendritic cells, and neutrophils have been deeply discussed (128–132). Chia-Ing Jan et al. designed and tested a CAR strategy to target HLA-G in solid tumors, and the results showed that HLA-G CAR-transduced NK cells effectively cytolyzed breast, brain, pancreatic and ovarian cancer cells *in vitro* and resulted in reduced xenograft tumor growth with extended median survival in orthotopic mouse models (133). In our study, we found that HLA-G desensitizes breast cancer cells to trastuzumab by binding to the NK-cell receptor KIR2DL4 and the blockade of HLA-G/KIR2DL4 axis improves the vulnerability of HER2-positive breast cancer to trastuzumab treatment *in vivo* (134).

## 4 Checkpoint immunotherapy based on macrophage

As an essential innate immune population, macrophages are also important components of the tumor microenvironment (TME). Tumor-associated macrophages (TAMs) have been found to be the most abundant immune cell type in solid tumors and to play an important role in orchestrating the immunosuppressive mechanism of the TME (135). Macrophages are highly plastic and generally can be classified into two polarized cell types: classically activated M1 cells and alternatively activated M2 cells. M1 cells have an antitumor function with a proinflammatory phenotype, and M2 cells can promote tumor progression as immunosuppressive cells. The specific phenotype or polarization type a macrophage assumes is dependent on factors released from TME (136). Many studies have revealed that macrophages play key roles in homeostasis and tumor development; thus, they have been regarded as promising targets for immunotherapy in a variety of diseases.

### 4.1 PD-1

In addition to T cells and NK cells, PD-1 has been found to be expressed in macrophages, and its expression increases over time and with disease progression (137, 138). Previous studies focused on blockade of the PD-1/PD-L1 axis have demonstrated the promising role of PD-1 in rejuvenating T cells, but the influence of axis blockade on macrophages has not been fully revealed. A recent study has reported that the expression of PD-L1 on macrophages is correlated with clinical responses to anti-PD-L1 therapy; moreover, macrophage polarization can have an effect on the suppression of tumor metastasis (139). Genevieve P Hartley et al. used PD-L1 antibodies to treat mouse and human macrophages and found that the treatment increased spontaneous macrophage proliferation, survival and activation, as indicated by evidence including costimulatory molecule expression and cytokine production. In an *in vivo* model, the use of a PD-L1 antibody increased tumor infiltration by activated macrophages and triggered macrophage-mediated antitumor activity (140). On the other hand, macrophages may be regulators participating in the mechanism of PD1/PD-L1 treatment resistance. Arlauckas et al. found that PD-1^+^ CD8^+^ T cells bound PD-1 antibody in a transient period, and then the antibody was seized within minutes from the T-cell surface by PD-1^-^ macrophages, which led to the failure of reactivation of exhausted T cells (141). Therefore, consideration of the macrophage effect and phenotype in checkpoint immunotherapy is very important.

### 4.2 CTLA-4

In a study analyzing the action of ipilimumab, a CTLA-4 blocking mAb, Emanuela Romano et al. found that unlike nonresponder patients, patients who respond to ipilimumab treatment display higher peripheral frequencies of nonclassical monocytes at baseline and enrichment of tumor-infiltrating CD68+CD16+ macrophages (142). Previously, Tyler R Simpson et al. explored the activity of an anti-CTLA-4 antibody in the treatment of metastatic melanoma and found that blocking CTLA-4 resulted in selective depletion of Treg cells within tumor lesions; remarkably, this depletion was dependent on Fcγ receptor-expressing macrophages in the TME (143). TAM-mediated elimination of anti-CTLA4-sensitized Tregs resulted in effective antitumor immunity. These results suggest that macrophages in the tumor microenvironment may contribute to the action of anti-CTLA-4 antibodies in tumor treatment.

### 4.3 CD47-SIRPα

Signal regulatory protein alpha (SIRPα) is a receptor expressed on macrophages that can interact with CD47, which is upregulated on some tumor cells, and thus transmit a “don’t eat me” signal. This is a strategy that is used by tumor cells to avoid phagocytosis. Based on this, anti-CD47 antibodies or engineered SIRPα-Fc fusion proteins have been used to prevent the immunosuppressive signal and restore macrophage phagocytic ability. Inhibition of the CD47/SIRPα axis can reduce tumor size and metastasis in many tumor models (144, 145). In clinical trials, anti-CD47 antibodies such as Hu5F9-G4 and CC-90002 and engineered high-affinity SIRPα and SIRPα-Fc fusion proteins (ALX148 and TTI-621) have been investigated for their therapeutic effects. However, this strategy has a defect: because of the ubiquitous expression of CD47 on red blood cells, anti-CD47 therapy can also lead to transient anemia (146). However, an alternative method has emerged involving a bispecific antibody that can target CD47 and tumor-associated antigens at the same time (147). Moreover, researchers have found that SIRPα is upregulated in NK cells upon IL-2 stimulation and interacts with target cell CD47 in a threshold-dependent manner. SIRPα deficiency or antibody blockade increases the killing capacity of NK cells, so disruption of the SIRPα-CD47 immune checkpoint may augment NK-cell antitumor responses, and elevated expression of CD47 may prevent NK-cell-mediated killing of allogeneic and xenogeneic tissues (148).

### 4.4 SFRs

In the study of phagocytic responses of different tumor cells to phagocytic cells when using SIRPα-CD47 blackade, Chen et al. found that phagocytosis of haematopoietic tumor cells during SIRP*α*–CD47 blockade was strictly dependent on SLAM (signalling lymphocytic activation molecule) family receptors (SFRs) *in vitro* and *in vivo* in mouse model. As the same results obtained in mouse, they also confirmed that this dependence required SLAMF7 (CD319 or CRACC), a SLAM family member which expressed on macrophages and tumor cell targets in human cells. Unlike other SLAM receptors, whose phagocytosis function are dependent on signalling lymphocyte activation molecule-associated protein (SAP) adaptors, SLAMF7 depended on its interaction with integrin Mac-1 and signals involving immunoreceptor tyrosine-based activation motifs. What counts is, their findings suggest that maybe the SIRP*α*–CD47 blockade therapy are more effective in patients with SLAMF7 expressing (149). Recently, Li et al. reported a critical role of the other two members of SFRs, SLAMF3 and SLAMF4, in constraining macrophage phagocytosis. Because of their ubiquitous expression on hematopoietic cells, the authors knockout SLAMF3 and SLAMF4 and found that the SFRs deficiency increased the ability of macrophages to phagocytose hematopoietic cells. In mouse model, the SFRs knockout lead to hematopoietic tumor rejection. Importantly, in CAR-macrophage therapy of hematopoietic cancer, the SFRs deletion also enhanced the efficacy. Together, their finding pointing to a potential therapeutic target for hematopoietic cancers (150).

### 4.5 Clever-1

The full name of Clever-1 is common lymphatic endothelial and vascular endothelial receptor-1, and it is also called Stabilin-1 or Feel-1. It is a conserved, multifunctional adhesion and scavenger receptor that is expressed by some endothelial cells and immunosuppressive macrophages and TAMs. Recent studies have found that Clever-1 can promote tumor progression (151–153). Miro Viitala et al. found that removal of Clever-1 from macrophages can significantly impair tumor growth in multiple solid tumor models, and a lack of Clever-1 in macrophages is associated with an increasingly immunostimulatory phenotype and enhanced signaling through the inflammatory mTOR pathway. Then, anti-Clever-1 treatment displays outcomes comparable to those of PD-1 blockade, implying Clever-1 as a novel target in clinical cancer evaluation and immunotherapy (154).

### 4.6 CD24/Siglec-10

CD24, a surface protein that is also called heat-stable antigen (HSA) or small cell lung carcinoma cluster 4 antigen, can interact with Siglec-10 and elicit inhibitory signals. CD24 has been reported to be expressed in several solid cancer cells (155, 156). As a member of the Siglec family, siglec-10 bears an ITIM within its cytoplasmic domain and can conduct inhibitory signals. Amira A Barkal et al. reported that many tumors overexpress CD24 and that TAMs express high levels of siglec-10. They found that the phagocytosis of all CD-24-expressing human tumors tested was augmented when CD24 or Siglec-10 was ablated genetically or when an antibody was used to block the CD24/Siglec-10 axis. In an *in vivo* study, ablation and blockade of CD24 resulted in both a macrophage-dependent reduction in tumor growth and extension of survival. These findings reveal the CD24/Siglec-10 axis as a promising new therapeutic target in cancer immunotherapy (157).

## 5 Checkpoint immunotherapy based on DCs

### 5.1 LAG-3

LAG-3 was found to be expressed on a subset of circulating human plasmacytoid dendritic cells (pDCs), and its interaction with MHC II can induce TLR-independent activation of pDCs with limited IFN-α and enhanced IL-6 production. The same study also found LAG-3+ pDCs in melanoma-invaded lymph nodes that were IL-6 positive. These results suggest that activation of pDCs induced by LAG-3 could be involved in creating a suppressive environment in tumor sites (158).

### 5.2 TIM-3

In addition to T cells, TIM-3 is expressed by multiple other cell types, including dendritic cells, and the expression of TIM-3 may inhibit nucleic acid sensing through TLRs (159). A recent study identified TIM-3, which is expressed by intratumoral CD103^+^ dendritic cells, as a target for therapy in a murine model of breast cancer. In that study, the use of an anti-TIM-3 antibody improved the response to paclitaxel chemotherapy in models of triple-negative and luminal B disease, with no evidence of toxicity. Anti-TIM-3 antibody administration led to enhanced granzyme B expression by CD8^+^ T cells and increased CXCR3 chemokine ligand expression by tumor conventional dendritic cells (160). Karen O. Dixon et al. demonstrated that loss of TIM-3 on dendritic cells, but not on CD4^+^ or CD8^+^ T cells, promotes strong antitumor immunity; moreover, it prevents dendritic cells from expressing a regulatory program and facilitates the maintenance of CD8^+^ effector and stem-like T cells. Conditional deletion of TIM-3 in dendritic cells leads to increased accumulation of reactive oxygen species, resulting in NLRP3 inflammasome activation, which underscores the potential of TIM-3 blockade for promoting antitumor immunity by regulating inflammasome activation (161). Overall, the immunomodulatory function mediated by TIM-3 is complex because of the broad expression of TIM-3 in different immune cells and the different interactions of this molecule with multiple ligands. Although promising therapeutic results have been reported in patients with anti-PD1-refractory disease in whom TIM-3 is co-blocked with other checkpoint receptors, the potential of TIM-3 as a drug target in different pathological conditions needs further study (162).

### 5.3 PD-L1

In a study investigating the anti-tumor mechanism of anti–PD-1 or PD-L1 antibodies, Mayoux et al. characterized various ligands on the surface of dendritic cells and found that PD-L1 is expressed much more abundantly than B7.1 on peripheral and tumor-associated dendritic cells in patients with cancer. PD-L1 expressed on dendritic cells can bind B7.1 on the same cell. This binding potentially prevent PD-1 ligation on T cells or B7.1 ligation of its partner CD28. Blocking PD-L1 on DCs relieves B7.1 sequestration in cis by PD-L1, which allows the B7.1/CD28 interaction to enhance T cell priming. This finding revealed that PD-L1 blockade reinvigorates DC function to generate potent anticancer T cell immunity (163).

## 6 Discussion

**Figure 2:**
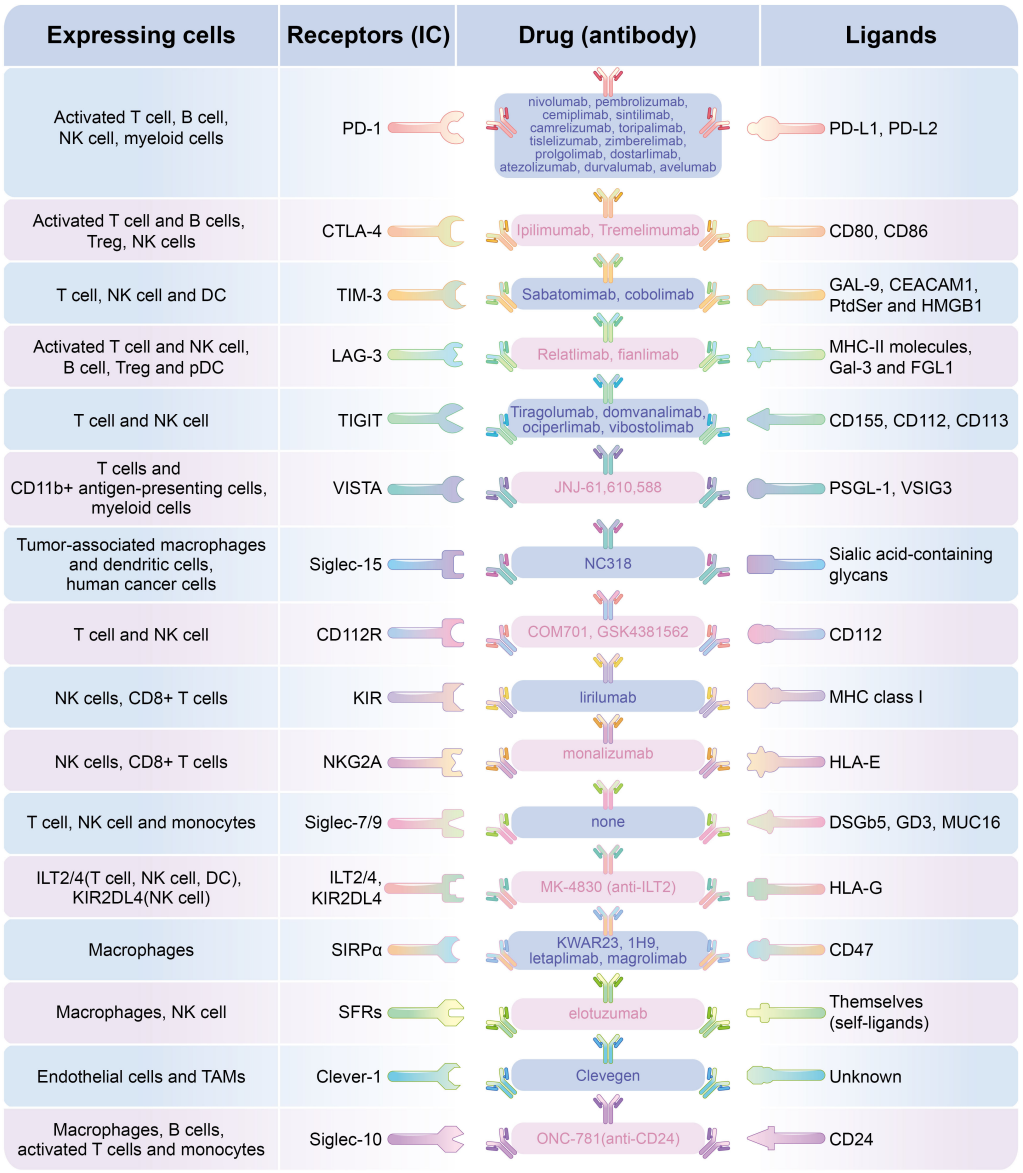
Different ICs expressing on different lymphocytes and and the targeted blocking antibody.

Complex communications between different cells and between cells and their surrounding microenvironment manipulate tumor oncogenesis and progression. In the tumor microenvironment, tumor cells create favorable conditions for cancer progression and avoid immunological surveillance through many strategies. For example, they can reduce neoantigen expression and alter the expression of immunoregulatory molecules on themselves. In addition, other extrinsic factors in the TME, such as the composition of tumor-infiltrating lymphocytes (TILs) and the inhibitory receptors expressed by TILs, all determine the ultimate direction of tumor development (164). Based on this, cancer immunotherapy, which mainly includes adoptive cell transfer (ACT) and immune checkpoint (IC) inhibitor (ICI) therapy, has revolutionized cancer treatment. In this review, we mainly discussed the diversity of immune checkpoints which have been found to be widely distributed in different immune cells and play different regulatory role. With the research and application of immunotherapy based on immune checkpoints in various malignant tumors (**Figure 2** and **Table 1**), their anti-tumor prospects are exciting, but there are still many problems in clinical application. The first question is that most patients exhibit primary or acquired resistance, one possible reason is due to compensatory mechanisms, such as upregulation of alternative immune checkpoints in addition to the widely noted PD-1 and CTLA-4, such as TIM-3 and VISTA, or the influence of many factors in the tumor immune microenvironment on T cell function. To explore the diversity of IC and their different effects on different lymphocytes, as well as to identify new therapeutic targets in the tumor microenvironment, will help guide the application of multi-ICI combination in clinical tumor therapy. To explore the key immunosuppressive pathways in different tumor types and different patient populations is particularly important for selecting the right immunotherapy (165). In addition, studies have found that in some refractory tumors (immunologically cold), the combination of antibodies targeting reverse inhibitory immune microenvironment and anti-PD-1 antibody can often improve the therapeutic effect (154). The second question, there is currently no effective method to distinguish ICI responders from non-responders. But with further research, the discovery of more immune checkpoints and their ligands may help predict the PD-1 therapeutic response in some tumors. For example, it has been found that the expression of inhibitory ligands for Tim-3 and Lag-3 on GC cells serve as potential biomarkers to predict the response to anti-PD-1 therapy and the combinatorial immunotherapy with ICIs targeting for PD-1, Tim-3, and Lag-3 has a therapeutic potential for GC patients (70). Third question, the irAEs present in the clinical ICI treatment is a huge problem, including systemic toxicity, dermotoxicity, gastrointestinal toxicity, endocrine toxicity, pulmonary toxicity, rheumatism, nervous system toxicity, ocular toxicity, renal toxicity, cardiotoxicity and hematological toxicity (166). These side effects will seriously affect the therapeutic effect and prognosis of patients. What’s worse, studies have found that the combined use of ICI may lead to a higher incidence of irAEs than single ICI therapy, depending on the type of malignancy and ICI used (167). At present, the cause of irAEs is not clear, but possible causes include non-specific immune stimulation of organ-specific inflammation, tissue damage and autoimmunity (168). Studies have found that the use of some immune checkpoint antibodies can affect the normal immune function of other normal tissues at the same time. For example, the use of CTLA-4 monoclonal antibodies can simultaneously produce an inhibitory effect on Treg cells expressing CTLA-4, leading to the destruction of immune tolerance, and thus an increase in the frequency and severity of irAEs was observed in some cases (169, 170). In view of the wide expression of immune checkpoints in various lymphocytes listed in this paper and the wide distribution of the same immune checkpoint in different lymphocytes (**Table 1**), the immune response caused by the application of ICI in the whole immune system should be fully considered. It will be an urgent topic for ICI treatment in the future to consider avoiding severe irAEs caused by the breakdown of autoimmune balance while achieving good anti-tumor efficacy.

**Table 1:** The description of IC molecules、targeted monoclonal antibody drugs and indications.

IC	Expressing cells	Targeted monoclonal antibody	Indications
PD-1	Activated T cell, B cell, NK cell, myeloid cells	nivolumab, pembrolizumab, cemiplimab, sintilimab, camrelizumab, toripalimab, tislelizumab, zimberelimab, prolgolimab, dostarlimab	Melanoma, NSCLC, RCC, HCC, Hodgkin's lymphoma, primary mediastinal large B cell lymphoma, SCC of the head and neck, urothelial carcinoma, gastric cancer, solid tumors with high MSI, or MRD, Cutaneous squamous cell carcinoma
PD-L1	various malignancies, dendritic cells	atezolizumab durvalumab avelumab	NSCLC, urothelial carcinoma, bladder cancer, Merkel cell carcinoma
CTLA-4	Activated T cell and B cells, Treg, NK cells	Ipilimumab	malignant melanoma, NSCLC, mesothelioma, prostate cancer, breast cancer, urothelial cancer
		Tremelimumab	
Tim-3	T cell, NK cell and DC	Sabatomimab	Advanced Malignancies
		cobolimab	
LAG-3	Activated T cell and NK cell, B cell, Treg and pDC	relatlimab	unresectable or metastatic melanoma
		fianlimab	
TIGIT	T cell and NK cell	tiragolumab	Melanoma, liver cancer, cervical cancer, prostate cancer, ESCC, breast cancer, NSCLC, NHL/DLBCL/B-cell malignancies
		domvanalimab	
		ociperlimab	
		vibostolimab	
VISTA	T cells and CD11b+ antigen-presenting cells, myeloid cells	JNJ-61,610,588	NSCLC, small-cell lung cancer, head and neck, pancreatic, colorectal, cervical cancer
Siglec-15	tumor-associated macrophages and dendritic cells, human cancer cells cells	NC318	advanced solid tumors
CD112R	T cell and NK cell	COM701	Breast cancer, Melanoma, pancreatic cancer
		GSK4381562	
KIR	NK cells, CD8+ T cells	lirilumab	MM, AML, relapsed/refractory lymphomas
NKG2A	NK cells, CD8+ T cells	monalizumab	oral squamous cell carcinoma, gynecological malignancies, relapsed hematological malignancies
Siglec-7/9	T cell, NK cell and monocytes	none	NSCLC, ovarian, colorectal cancers, melanoma
HLA-G	various malignancies	none	Breast cancer
ILT2/4,KIR2DL4	ILT2/4(T cell, NK cell, DC), KIR2DL4(NK cell)	MK-4830 (anti-ILT2)	solid malignancies and hematological malignancies
SIRPα	macrophages	KWAR23	Burkitt's lymphoma, Melanoma
		1H9	
CD47	many tumor cells	letaplimab	Melanoma, AML stem cells, Breast cancer
		magrolimab	
SFRs	Macrophages, NK cell	elotuzumab	MM
Clever-1	Endothelial cells and TAMs	Clevegen	cutaneous and uveal melanoma, hepatobiliary, pancreatic, ovarian, oestrogen-receptor-positive breast, colorectal, gastric, gallbladder cancer and cholangiocarcinoma
Siglec-10	Macrophages, B cells, activated T cells and monocytes	ONC-781(anti-CD24)	Advanced Solid Tumors, Unresectable or metastatic melanoma, Resected HCC

HCC, hepatocellular carcinoma; MRD, minimal residual disease; MSI, microsatellite instability; NSCLC, nonsmall-cell lung carcinoma; RCC, renal cell carcinoma; SCC, squamous cell carcinoma. MM, multiple myeloma; AML, acute myeloid leukemia; ESCC, esophageal squamous cell carcinoma.

## Author contributions

Conceptualization, funding acquisition: ZG. Writing original draft, review and editing: GZ、RZ and A-GY. All authors contributed to the article and approved the submitted version.
